# Potential asthma biomarkers identified by nontargeted proteomics of extracellular vesicles in exhaled breath condensate

**DOI:** 10.1016/j.jacig.2025.100432

**Published:** 2025-01-30

**Authors:** Reina Hara, Yoshito Takeda, Takatoshi Enomoto, Hanako Yoshimura, Makoto Yamamoto, Satoshi Tanizaki, Yuya Shirai, Takahiro Kawasaki, Mana Nakayama, Saori Amiya, Yuichi Adachi, Yoshimi Noda, Takayuki Niitsu, Ryuya Edahiro, Moto Yaga, Yuki Hosono, Maiko Naito, Kentaro Masuhiro, Yujiro Naito, Takayuki Shiroyama, Kotaro Miyake, Kiyoharu Fukushima, Shohei Koyama, Kota Iwahori, Haruhiko Hirata, Izumi Nagatomo, Yusuke Kawashima, Mari Nogami-Itoh, Atsushi Kumanogoh

**Affiliations:** a Department of Respiratory Medicine and Clinical Immunology, Osaka University Graduate School of Medicine, Suita, Osaka, Japan; b Department of Applied Genomics, Kazusa DNA Research Institute, Kisarazu, Japan; c Compound Library Screening Center Graduate School of Pharmacological Sciences, Osaka University, Suita, Osaka, Japan; d Department of Immunopathology, Immunology Frontier Research Center, Osaka University, Suita, Osaka, Japan; e Center for Advanced Modalities and DDS, Osaka University, Suita, Osaka, Japan; f Center for Infectious Diseases for Education and Research, Osaka University, Suita, Osaka, Japan; g Integrated Frontier Research for Medical Science Division, Institute for Open and Transdisciplinary Research Initiatives, Osaka University, Suita, Osaka, Japan; h Japan Agency for Medical Research and Development–Core Research for Evolutional Science and Technology, Osaka University, Suita, Osaka, Japan

**Keywords:** Exhaled breath condensate, proteomics, bioinformatics, biomarker, exosome, extracellular vesicles, bronchial asthma, COPD, phenotype, endotype

## Abstract

**Background:**

Bronchial asthma (BA) and chronic obstructive pulmonary disease (COPD) are often misdiagnosed or undiagnosed, highlighting the need for more noninvasive and accessible diagnostic tools. Although exhaled breath condensate (EBC) is recognized as a biomarker resource for respiratory diseases, nontargeted proteomics of extracellular vesicles (EVs) in EBC has not been explored.

**Objective:**

Our aim was to identify protein signatures in EBC-derived EVs (EBC-EVs) and potential biomarkers for BA and COPD.

**Methods:**

EBC-EVs were isolated from 8 patients with BA, 5 patients with COPD, and 9 healthy controls by using the phosphatidylserine affinity method. The isolated EBC-EVs were analyzed by using data-independent acquisition proteomics to identify differentially expressed proteins (DEPs) and their associations with clinical parameters.

**Results:**

Overall, 2524 proteins were identified. In the patients with BA, 20 proteins were upregulated, and 34 were downregulated. In the patients with COPD, 46 proteins were upregulated and 67 were downregulated. Although the enriched pathways and protein networks showed similarities between BA and COPD, they also indicated distinct pathophysiologic differences. In all, 5 BA-DEPs and 2 COPD-DEPs correlated with clinical parameters. For BA, S100 calcium-binding protein P levels were inversely correlated with FEV_1_ value, and ribosomal protein S10 levels were inversely correlated with blood eosinophil count. Clathrin heavy chain 2 correlated with serum IgE levels. For COPD, 14-3-3 protein theta and galectin-related protein showed positive and negative correlations with FEV_1_ value, respectively.

**Conclusions:**

Proteomics of EBC-EVs has enabled the identification of potential diagnostic biomarkers for BA and COPD. “Breathomics” of EBC-EVs offers a promising noninvasive approach for diagnosis and phenotyping of respiratory diseases.

## Introduction

Bronchial asthma (BA) is characterized by chronic airway inflammation, leading to variable respiratory symptoms and expiratory airflow limitation and affecting 300 million people worldwide.^1^ It shares common symptoms and pathogenesis with chronic obstructive pulmonary disease (COPD), a chronic inflammatory lung disease that is typically caused by smoking and results in obstructed airflow.^2^ Despite the benefits of early diagnosis and treatment in reducing hospital visits and symptom worsening for both BA and COPD, many patients remain undiagnosed.^3^ The diagnosis of BA is supported by markers reflecting type 2 inflammation, such as fractional exhaled nitric oxide (F_ENO_) level, blood eosinophil count, and serum IgE level.^4^ Although F_ENO_ level is easy to measure, it primarily reflects IL-13–mediated type 2 airway inflammation, providing a limited diagnostic perspective on the disease.^5^ Therefore, the development of noninvasive novel biomarkers would significantly improve both the diagnosis and understanding of the pathophysiology of BA.

Exhaled breath condensate (EBC) can be collected easily and noninvasively, and it contains various volatile and nonvolatile compounds from the lower airways, reflecting local pathophysiology such as airway inflammation.^6^ Although EBC has long been a focus of biomarker research, the low concentration of the contained compounds has posed a significant challenge.

Extracellular vesicles (EVs) are small membrane-bound vesicles that are released from cells containing biomolecules related to physiologic functions. Consequently, they have become a focus of biomarker research.^7^ Sinha et al reported that 11 microRNAs (miRNAs) from EBC-derived EVs (EBC-EVs) exhibited differential expression in BA.^8^ For serum, EVs have proved useful in identifying a larger number of proteins by removing contaminants,^9^ and EV protein biomarkers have been reported for inflammatory lung diseases, including asthma.^9^, ^10^, ^11^ However, research on EV proteins remains limited owing to their small quantities, which makes them difficult to quantify.^12^

With advances in technology, sensitive and comprehensive analysis has enabled the identification of EBC biomarkers for respiratory diseases, particularly miRNA and metabolite biomarkers. Although miRNA profiles and their usefulness in diagnosis and disease monitoring have been described for BA,^13^^,^^14^ clinically useful protein biomarkers in EBC-EVs, including those for BA, have been scarce in EBC research, despite the diversity of proteins and their closer link to phenotypes.^15^ To date, no studies have included nontargeted proteomics analysis of EBC for BA or COPD, and proteomics analysis of EBC-EVs has never been performed. Therefore, we aimed to identify novel diagnostic biomarkers for BA and COPD through the latest nontargeted proteomics of EBC-EVs.

## Results and discussion

**Fig 1:**
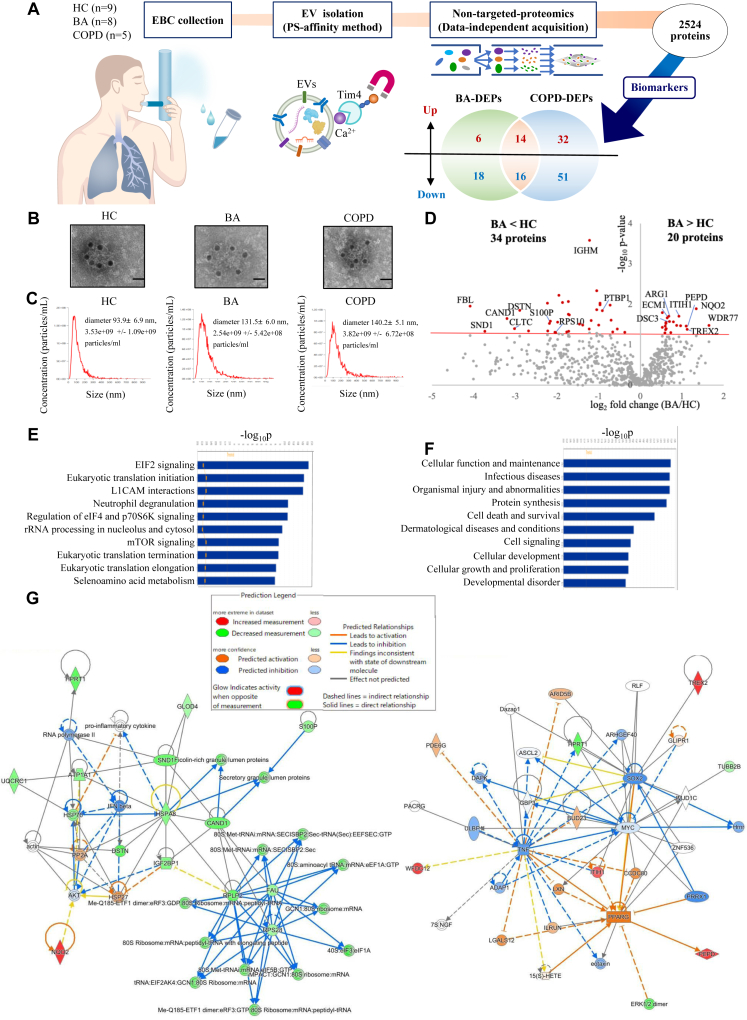
Study overview and DEPs in patients with BA or COPD. **A,** Study overview. **B,** Transmission electron microscope images of EBC-EVs, immunogold-labeled with CD63 antibody. **C,** Sizes and numbers of EBC-EVs confirmed by nanoparticle tracking analysis. **D,** All 711 proteins adopted for significance testing between the patients with BA and the HCs (*P* < .050). **E,** Top 10 canonical pathways for BA-DEPs. **F,** Top 10 diseases and functions for BA-DEPs. **G,** Protein-protein interactions of BA-DEPs. Scale bars = 100 nm.

EBC was collected during normal breathing for 10 to12 minutes by using an R-Tube (Respiratory Research Inc, Austin, Tex), after which the samples were immediately frozen at −80°C. A summary of the study is shown in Fig 1, *A*, and patient characteristics are shown in Table I. Transmission electron microscopy confirmed expression of the EV marker CD63 on the membrane surface of isolated EBC-EVs (Fig 1, *B*).^16^ Nanoparticle tracking analysis revealed that the diameters of EBC-EVs were less than 200 nm, with no differences in the number or size of EBC-EV particles observed between the BA, COPD, and healthy control (HC) groups (Fig 1, *C*).

**Table I:** Characteristics of HCs, patients with BA, and patients with COPD

Characteristic	HC group (n = 9)	BA group (n = 8)	COPD group (n = 5)	*P* value
Age (y), mean ± SD	63.8 ± 11.4	65 ± 8.39	71 ± 4.89	.164
Male sex, no (%)	5 (55)	2 (25)	5 (100)	**.025**
BMI, kg/m^2^, mean ± SD	22.6 ± 3.02	23.6 ± 3.01 (n = 7)	25.3 ± 2.7	.26
Smoking status, no.	Former 2/never 7	Former 1/never 7	Former 5/never 0	**.00027**
Pack years, no.	45	1.5	74.2 ± 57	.22
ACT scores, median		20.5		
GINA step 1/2/3/4/5, no.		0/0/1/2/5		
BA medication, no.				
ICS/LABA		3		
ICS/LABA/LTRA		1		
ICS/LABA/LAMA/LTRA		4		
GOLD stage I/II/III/IV, no.			0/5/0/0	
COPD medication, no.				
LAMA/LABA/ICS			1	
LAMA			2	
ICS/LABA			1	
None			1	
F_ENO_ level, ppb (n=14)		99.4 ± 60.8		
Blood eosinophil count (/μL), mean ± SD		323.4 ± 239	142.6 ± 104	**.0035**
Serum IgE level (kU/mL), median [IQR]		135.7 [82.5-635.6]	220.1	**<.0001**
FEV_1_ value (mL), mean ± SD		2023 ± 873	1940 ± 96.4	.851
%FEV_1_ value, mean ± SD		82.8 ± 14.8	68.7 ± 4.83	.0591
EBC volume (μL), mean ± SD	1250 ± 378	1300 ± 149	1571 ± 531	.312

Boldface indicates statistical significance.

*BMI*, Body mass index; *GINA*, Global Initiative for Asthma; *GOLD*, Global Initiative for Chronic Obstructive Lung Disease; *ICS*, inhaled corticosteroid; *IQR*, interquartile range; *LABA*, long-acting β-agonist; *LAMA*, long-acting antimuscarinic agent; *LTRA*, leukotriene receptor antagonist.

Data-independent analysis (DIA) proteomics of EBC-EVs identified a total of 2524 proteins (Fig 1, *A*). To the best of our knowledge, this is the largest number of proteins identified from EBC reported to date. Following missing value imputation, 593 proteins were selected for further analysis. Tissue expression analysis identified 261 lung-associated proteins, which far exceeded the 14 saliva-associated proteins, thus suggesting that the impact of saliva contamination was minimal. A comparison between the BA and HC groups revealed differences in 54 proteins (*P* < .05), with the levels of 20 proteins increased and those of 34 proteins decreased in the BA group (Fig 1, *D* and Table II). Ingenuity Pathway Analysis (IPA) indicated that the altered pathways in BA were predominantly related to eukaryotic translation, such as eukaryotic initiation factor 2 signaling (Fig 1, *E*). Among these proteins, the mammalian target of the rapamycin (mTOR) signaling pathway has been reported to be significantly upregulated in the serum of patients experiencing an asthma attack,^17^ and inhibiting the mTOR pathway in asthmatic mice reduces asthma markers.^17^ Diseases and function analysis by IPA revealed enriched pathways in BA, such as “cell death and survival” (Fig 1, *F*). Protein-protein interactions of differentially expressed proteins (DEPs) in BA (BA-DEPs) suggested the involvement of proinflammatory cytokines and 40s ribosomal proteins in BA pathophysiology (Fig 1, *G*). Overall, the latest nontargeted proteomics enabled the characterization of EBC proteins from asthmatic individuals, demonstrating diagnostic potential.

**Table II:** DEPs in the BA or COPD groups versus in the HC group

Protein ID	Gene name	Protein name	vs HC *P* value	vs HC Fold change	Uncommon DEPs between BA and COPD groups
DEPs in the BA group					
Q9BQA1	*WDR77*	WD repeat domain 77	.033	3.1	
P16083	*NQO2*	Ribosyldihydronicotinamide dehydrogenase [quinone]	.012	2.5	
Q9BQ50	*TREX2*	Three prime repair exonuclease 2	.041	2.2	
P12955	*PEPD*	Xaa-Pro dipeptidase	.034	2.1	☑
P48637	*GSS*	Glutathione synthetase	.033	2.0	
P19827	*ITIH1*	Inter-alpha-trypsin inhibitor heavy chain H1	.019	1.9	
Q8WWY7	*WFDC12*	WAP 4-disulfide core domain protein 12	.033	1.8	
Q9H0W9	*C11orf54*	Ester hydrolase C11orf54	.026	1.7	☑
Q9UJ70	*NAGK*	N-acetyl-D-glucosamine kinase	.049	1.6	☑
Q14574	*DSC3*	Desmocollin-3	.026	1.6	☑
P05089	*ARG1*	Arginase-1	.019	1.6	
Q16610	*ECM1*	Extracellular matrix protein 1	.021	1.6	
P31944	*CASP14*	Caspase-14	.039	1.5	
P30086	*PEBP1*	Phosphatidylethanolamine-binding protein 1	.028	1.5	
Q96P63	*SERPINB12*	Serpin B12	.029	1.5	
O75223	*GGCT*	Gamma-glutamylcyclotransferase	.028	1.5	
Q5T749	*KPRP*	Keratinocyte proline-rich protein	.041	1.5	
Q08554	*DSC1*	Desmocollin-1	.050	1.5	☑
Q15828	*CST6*	Cystatin-M	.037	1.4	☑
P04040	*CAT*	Catalase	.016	1.4	
P61604	*HSPE1*	10 kDa heat shock protein, mitochondrial	.048	0.77	☑
P02749	*APOH*	Beta-2-glycoprotein 1	.040	0.68	☑
P07737	*PFN1*	Profilin-1	.047	0.63	☑
P51884	*LUM*	Lumican	.024	0.61	☑
P12277	*CKB*	Creatine kinase B-type	.010	0.60	☑
P26599	*PTBP1*	Polypyrimidine tract-binding protein 1	.014	0.53	☑
P27348	*YWHAQ*	14-3-3 protein theta	.009	0.51	
P01834	*IGKC*	Immunoglobulin kappa constant	.010	0.51	☑
Q08380	*LGALS3BP*	Galectin-3-binding protein	.032	0.50	
P01871	*IGHM*	Immunoglobulin heavy constant mu	.004	0.48	☑
Q9HC38	*GLOD4*	Glyoxalase domain-containing protein 4	.013	0.48	☑
P06748	*NPM1*	Nucleophosmin	.040	0.42	
P02679	*FGG*	Fibrinogen gamma chain	.032	0.38	☑
P05023	*ATP1A1*	Sodium/potassium-transporting ATPase subunit alpha-1	.031	0.36	
P00492	*HPRT1*	Hypoxanthine-guanine phosphoribosyltransferase	.018	0.30	
P61978	*HNRNPK*	Heterogeneous nuclear ribonucleoprotein K	.046	0.30	
P05387	*RPLP2*	Large ribosomal subunit protein P2	.008	0.30	
P78347	*GTF2I*	General transcription factor II-I	.017	0.30	
P35232	*PHB1*	Prohibitin 1	.047	0.29	
Q9NZI8	*IGF2BP1*	Insulin-like growth factor 2 mRNA-binding protein 1	.009	0.27	
P28482	*MAPK1*	Mitogen-activated protein kinase 1	.006	0.26	
Q9Y262	*EIF3L*	Eukaryotic translation initiation factor 3 subunit L	.027	0.25	
P06454	*PTMA*	Prothymosin alpha	.049	0.24	☑
P46783	*RPS10*	Small ribosomal subunit protein eS10	.048	0.24	
P25815	*S100P*	Protein S100-P	.025	0.22	☑
P11142	*HSPA8*	Heat shock cognate 71 kDa protein	.029	0.22	☑
P62857	*RPS28*	Small ribosomal subunit protein eS28	.045	0.21	
Q00610	*H2AZ1*	Clathrin heavy chain 1	.009	0.21	☑
P61221	*ABCE1*	ATP-binding cassette sub-family E member 1	.045	0.16	☑
P60981	*DSTN*	Destrin	.014	0.13	☑
P53675	*CLTC*	Clathrin heavy chain 2	.039	0.12	☑
Q86VP6	*CAND1*	Cullin-associated NEDD8-dissociated protein 1	.022	0.11	
Q7KZF4	*SND1*	Staphylococcal nuclease domain-containing protein 1	.046	0.074	☑
P22087	*FBL*	rRNA 2'-O-methyltransferase fibrillarin	.011	0.058	
DEPs in the COPD group					

Q9BQA1	*WDR77*	Methylosome protein WDR77	.046	3.2	
P16083	*NQO2*	Ribosyldihydronicotinamide dehydrogenase [quinone]	.0043	2.9	
Q9UHL4	*DPP7*	Dipeptidyl peptidase 2	.035	2.7	☑
P51688	*SGSH*	N-sulphoglucosamine sulphohydrolase	.047	2.5	☑
Q9BQ50	*TREX2*	Three prime repair exonuclease 2	.015	2.3	
Q02413	*DSG1*	Desmoglein-1	.042	2.1	☑
P16870	*CPE*	Carboxypeptidase E	.039	2.1	☑
Q15773	*MLF2*	Myeloid leukemia factor 2	.023	2.1	☑
Q5VVQ6	*YOD1*	Ubiquitin thioesterase OTU1	.035	2.1	☑
Q8WWY7	*WFDC12*	WAP four-disulfide core domain protein 12	.018	2.1	
P14923	*JUP*	Junction plakoglobin	.028	2.1	☑
Q7Z4W1	*DCXR*	L-xylulose reductase	.0076	2.0	☑
P13473	*LAMP2*	Lysosome-associated membrane glycoprotein 2	.043	1.9	☑
P0DP57	*SLURP2*	Secreted Ly-6/uPAR domain-containing protein 2	.015	1.9	☑
P48637	*GSS*	Glutathione synthetase	.025	1.9	
P05089	*ARG1*	Arginase-1	.012	1.9	
Q6NUJ1	*PSAPL1*	Proactivator polypeptide-like 1	.012	1.9	☑
Q3ZCW2	*LGALSL*	Galectin-related protein	.047	1.8	☑
Q9H0E2	*TOLLIP*	Toll-interacting protein	.022	1.8	☑
P19827	*ITIH1*	Inter-alpha-trypsin inhibitor heavy chain H1	.023	1.8	
Q8NDH3	*NPEPL1*	Probable aminopeptidase NPEPL1	.032	1.7	☑
Q02487	*DSC2*	Desmocollin-2	.030	1.7	☑
Q16610	*ECM1*	Extracellular matrix protein 1	.019	1.7	
Q13835	*PKP1*	Plakophilin-1	.013	1.7	☑
Q9NZH8	*IL36G*	IL-36 gamma	.041	1.7	☑
O95867	*LY6G6C*	Lymphocyte antigen 6 complex locus protein G6c	.021	1.7	☑
O43548	*TGM5*	Protein-glutamine gamma-glutamyltransferase 5	.018	1.6	☑
Q5T750	*KPLCE*	Protein KPLCE	.0076	1.6	☑
P49189	*ALDH9A1*	4-Trimethylaminobutyraldehyde dehydrogenase	.030	1.6	☑
Q5T749	*KPRP*	Keratinocyte proline-rich protein	.025	1.6	
P07355	*ANXA2*	Annexin A2	.015	1.6	☑
O75608	*LYPLA1*	Acyl-protein thioesterase 1	.036	1.6	☑
P49720	*PSMB3*	Proteasome subunit beta type-3	.032	1.6	☑
Q08188	*TGM3*	Protein-glutamine gamma-glutamyltransferase E	.011	1.6	☑
P56537	*EIF6*	Eukaryotic translation initiation factor 6	.038	1.6	☑
P35754	*GLRX*	Glutaredoxin-1	.034	1.5	☑
Q96P63	*SERPINB12*	Serpin B12	.021	1.5	
P32119	*PRDX2*	Peroxiredoxin-2	.034	1.5	☑
Q9BYJ1	*ALOXE3*	Hydroperoxide isomerase ALOXE3	.030	1.5	☑
O75223	*GGCT*	Gamma-glutamylcyclotransferase	.028	1.5	
P30086	*PEBP1*	Phosphatidylethanolamine-binding protein 1	.049	1.5	
P31944	*CASP14*	Caspase-14	.050	1.5	
P07384	*CAPN1*	Calpain-1 catalytic subunit	.041	1.5	☑
P22735	*TGM1*	Protein-glutamine gamma-glutamyltransferase K	.048	1.5	☑
Q92820	*GGH*	Gamma-glutamyl hydrolase	.012	1.5	☑
P04040	*CAT*	Catalase	.0093	1.4	
P05141	*SLC25A5*	ADP/ATP translocase 2	.049	0.35	☑
Q8IXJ6	*SIRT2*	NAD-dependent protein deacetylase sirtuin-2	.049	0.34	☑
P14625	*HSP90B1*	Endoplasmin	.042	0.33	☑
P06748	*NPM1*	Nucleophosmin	.016	0.32	
P17600	*SYN1*	Synapsin-1	.041	0.32	☑
P19338	*NCL*	Nucleolin	.028	0.31	☑
Q08380	*LGALS3BP*	Galectin-3-binding protein	.0097	0.31	
Q96DA0	*ZG16B*	Pancreatic adenocarcinoma upregulated factor	.045	0.31	☑
P62937	*PPIA*	Peptidyl-prolyl cis-trans isomerase A	.047	0.30	☑
P04114	*APOB*	Apolipoprotein B-100	.0059	0.29	☑
O43175	*PHGDH*	D-3-phosphoglycerate dehydrogenase	.023	0.28	☑
Q9NZI8	*IGF2BP1*	Insulin-like growth factor 2 mRNA-binding protein 1	.017	0.27	
P61981	*YWHAG*	14-3-3 protein gamma	.024	0.27	☑
Q9BPX5	*ARPC5L*	Actin-related protein 2/3 complex subunit 5-like protein	.0011	0.24	☑
P49411	*TUFM*	Elongation factor Tu, mitochondrial	.036	0.23	☑
P35232	*PHB1*	Prohibitin 1	.019	0.23	
P50213	*IDH3A*	Isocitrate dehydrogenase [NAD] subunit alpha, mitochondrial	.015	0.23	☑
P30101	*PDIA3*	Protein disulfide-isomerase A3	.032	0.23	☑
P21291	*CSRP1*	Cysteine and glycine-rich protein 1	.038	0.22	☑
P28482	*MAPK1*	Mitogen-activated protein kinase 1	.014	0.22	
P05387	*RPLP2*	Large ribosomal subunit protein P2	.0072	0.21	
P35268	*RPL22*	Large ribosomal subunit protein eL22	.033	0.20	☑
P27348	*YWHAQ*	14-3-3 protein theta	.016	0.20	
P26641	*EEF1G*	Elongation factor 1-gamma	.015	0.19	☑
P05023	*ATP1A1*	Sodium/potassium-transporting ATPase subunit alpha-1	.019	0.19	
P61978	*HNRNPK*	Heterogeneous nuclear ribonucleoprotein K	.0057	0.19	
P62081	*RPS7*	Small ribosomal subunit protein eS7	.031	0.19	☑
P62249	*RPS16*	Small ribosomal subunit protein uS9	.038	0.17	☑
P61088	*UBE2N*	Ubiquitin-conjugating enzyme E2 N	.032	0.17	☑
P00492	*HPRT1*	Hypoxanthine-guanine phosphoribosyltransferase	.0084	0.17	
P08238	*HSP90AB1*	Heat shock protein HSP 90-beta	.022	0.16	☑
P78347	*GTF2I*	General transcription factor II-I	.016	0.16	
Q16629	*SRSF7*	Serine/arginine-rich splicing factor 7	.004	0.15	☑
P07910	*HNRNPC*	Heterogeneous nuclear ribonucleoproteins C1/C2	.032	0.15	☑
Q15084	*PDIA6*	Protein disulfide-isomerase A6	.010	0.14	☑
Q9P2R7	*SUCLA2*	Succinate--CoA ligase [ADP-forming] subunit beta, mitochondrial	.046	0.14	☑
P30041	*PRDX6*	Peroxiredoxin-6	.0035	0.14	☑
P62857	*RPS28*	Small ribosomal subunit protein eS28	.0064	0.14	
Q9Y262	*EIF3L*	Eukaryotic translation initiation factor 3 subunit L	.013	0.13	
P46783	*RPS10*	Small ribosomal subunit protein eS10	.011	0.13	
P62826	*RAN*	GTP-binding nuclear protein Ran	.022	0.13	☑
Q15366	*PCBP2*	Poly(rC)-binding protein 2	.012	0.13	☑
P07237	*P4HB*	Protein disulfide-isomerase	.0038	0.12	☑
P18124	*RPL7*	Large ribosomal subunit protein uL30	.019	0.12	☑
P27797	*CALR*	Calreticulin	.043	0.12	☑
P62424	*RPL7A*	Large ribosomal subunit protein eL8	.050	0.10	☑
P22314	*UBA1*	Ubiquitin-like modifier-activating enzyme 1	.022	0.095	☑
Q07020	*RPL18*	Large ribosomal subunit protein eL18	.031	0.094	☑
P38606	*ATP6V1A*	V-type proton ATPase catalytic subunit A	.028	0.090	☑
O75367	*MACROH2A1*	Core histone macro-H2A.1	.0066	0.089	☑
P62888	*RPL30*	Large ribosomal subunit protein eL30	.046	0.078	☑
P45974	*USP5*	Ubiquitin carboxyl-terminal hydrolase 5	.0029	0.073	☑
Q96QK1	*VPS35*	Vacuolar protein sorting-associated protein 35	.018	0.069	☑
Q01082	*SPTBN1*	Spectrin beta-chain, nonerythrocytic 1	.048	0.069	☑
P08133	*ANXA6*	Annexin A6	.015	0.068	☑
O43707	*ACTN4*	Alpha-actinin-4	.034	0.067	☑
P22087	*FBL*	rRNA 2'-O-methyltransferase fibrillarin	.019	0.058	
Q86VP6	*CAND1*	Cullin-associated NEDD8-dissociated protein 1	.0027	0.052	
Q02790	*FKBP4*	Peptidyl-prolyl cis-trans isomerase FKBP4	.048	0.051	☑
P43487	*RANBP1*	Ran-specific GTPase-activating protein	.015	0.051	☑
Q9Y490	*TLN1*	Talin-1	.029	0.049	☑
P17987	*TCP1*	T-complex protein 1 subunit alpha	.015	0.046	☑
P51991	*HNRNPA3*	Heterogeneous nuclear ribonucleoprotein A3	.043	0.046	☑
O75531	*BANF1*	Barrier-to-autointegration factor	.01	0.037	☑
P61313	*RPL15*	Large ribosomal subunit protein eL15	.029	0.033	☑
Q14247	*CTTN*	Src substrate cortactin	.00063	0.033	☑
P49368	*CCT3*	T-complex protein 1 subunit gamma	.013	0.021	☑

All proteins were quantified by 2 or more unique peptides. *P* < .050.

**Fig 2:**
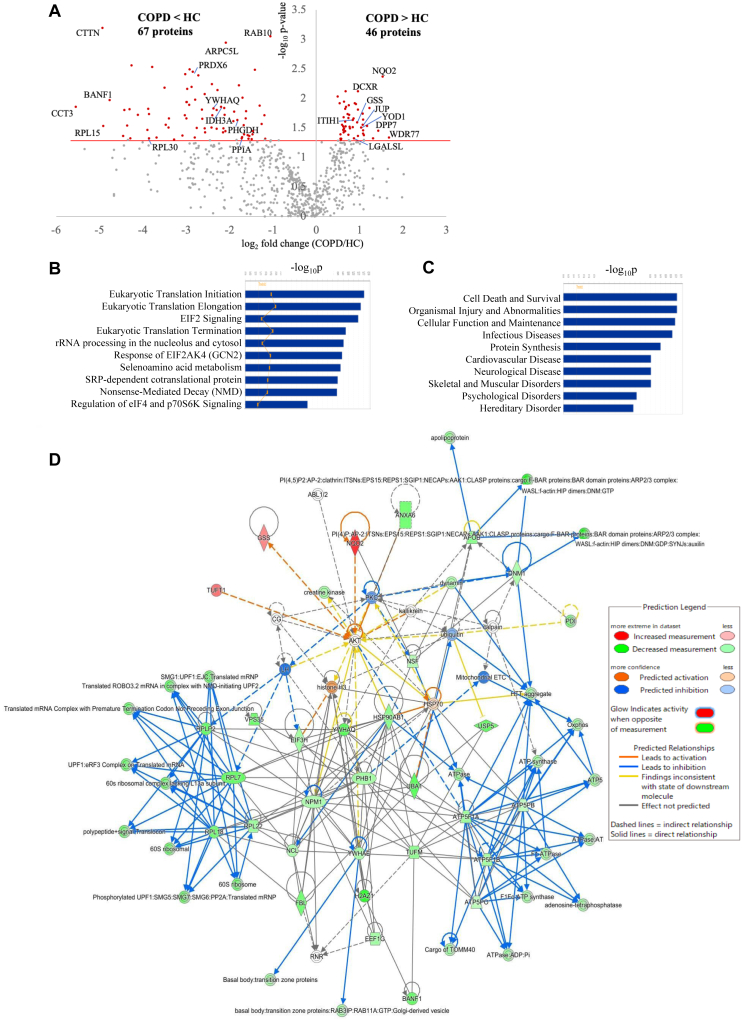
**A,** All 711 proteins adopted for significance testing between the COPD and HC groups (*P* < .050). **B,** Top 10 canonical pathways for COPD-DEPs. **C,** Top 10 diseases and functions for COPD-DEPs. **D,** Protein-protein interactions of COPD-DEPs.

Next, a comparison between the COPD and HC groups revealed 113 DEPs (COPD-DEPs), with 46 upregulated and 67 downregulated (*P* < .05 [Fig 2, *A* and Table II]). Altered pathways identified by IPA are shown in Fig 2, *B* and *C*. Protein-protein interactions of COPD-DEPs suggested changes in the expression of 60s ribosomal proteins (Fig 2, *D*). From the perspective of differentiating BA and COPD, 24 of the BA-DEPs (44%) were unique when compared with the COPD-DEPs (Fig 1, *A*). Interestingly, although BA and COPD shared similar altered canonic pathways, the diseases and functions analysis revealed different top-ranking categories for the 2 conditions. COPD notably featured pathways such as “cardiovascular disease” and “skeletal and muscular disorders,” which may reflect frailty and systemic inflammation. EBC-EV biomarkers have the potential to aid in the differential diagnosis of BA and COPD by complementing clinical assessments based on patient history and symptoms. Furthermore, the exhaled breath proteomic signature indicates both similarities and differences in the pathophysiology of BA and COPD.

**Fig 3:**
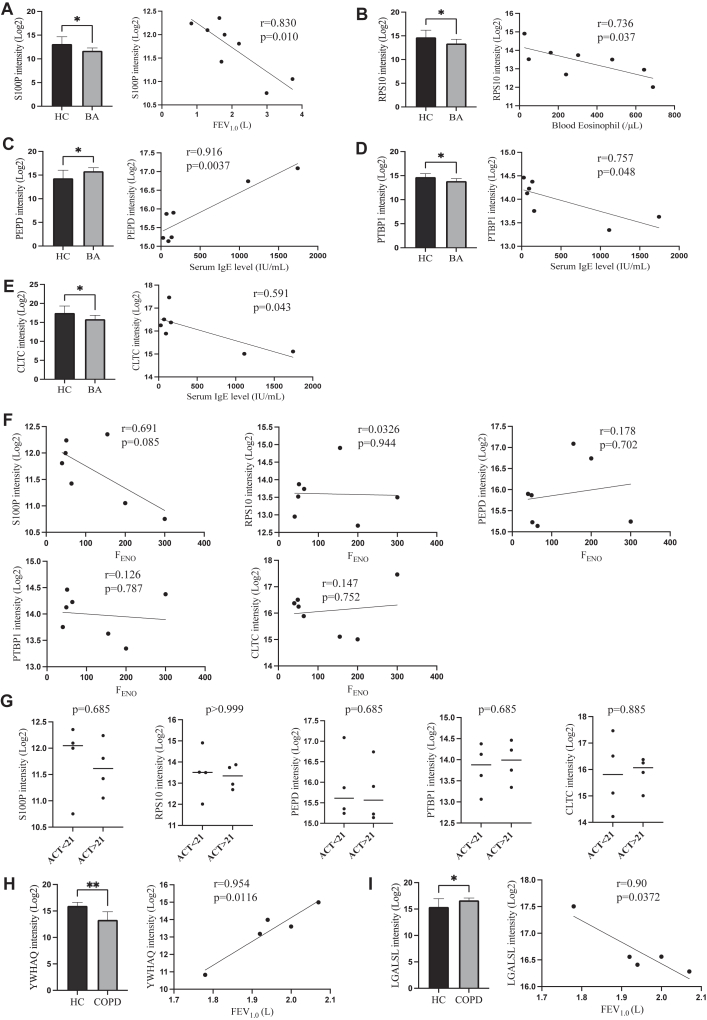
Significance tests performed on BA-DEPs or COPD-DEPs and correlation between BA-DEPs or COPD-DEPs and clinical parameters. **A,** Analysis of S100P. **B,** Analysis of RPS10. **C,** Analysis of PEPD. **D,** Analysis of PTBP1. **E,** Analysis of CLTC. **F,** Correlation between BA-DEPs and F_ENO_ level. **G,** Significance tests for BA-DEPs by Asthma Control Test (ACT) scores. **H,** Analysis of YWHAQ. **I,** Analysis oc LGALSL. *r* indicates Spearman rank correlation coefficient. ∗*P* < .050; ∗∗*P* = .010.

Furthermore, to assess the clinical potential of these biomarkers, we examined the correlation between the intensity of 54 BA-DEPs and the following clinical parameters: respiratory function, blood eosinophil count, serum IgE level, F_ENO_ level, and Asthma Control Test score. Notably, S100 calcium-binding protein (S100P), which was downregulated in BA, was inversely correlated with FEV_1.0_ value and showed a tendency to inverse correlation with F_ENO_ level (Fig 3, *A*). S100P, a Ca^2+^ binding protein from the S100 family, is localized in the cytoplasm and nucleus of various cells and is involved in chronic inflammation and airway obstruction.^18^ S100P is expressed in the airway epithelium and activates mitogen-activated protein kinase and nuclear factor-κB signaling, forming a core protein network in BA.^19^ Meanwhile, ribosomal protein S10 (RPS10), which is downregulated in BA, was inversely correlated with blood eosinophil count (Fig 3, *B*). RPS10 is a ribonucleoprotein localized in the cytoplasm, which is widely expressed in epithelial and immune cells, and is a component of the 40S ribosomal subunit.^20^ Additionally, peptidase D (PEPD) levels were positively correlated with serum IgE levels, whereas polypyrimidine tract-binding protein 1 (PTBP1) and clathrin heavy chain 2 (CLTC) levels were inversely correlated with serum IgE levels (Fig 3, *C-E*). PEPD is an intracellular dipeptidase involved in collagen metabolism.^21^ PTBP1, which is widely expressed in epithelial cells and localized in the nucleoplasm, regulates T-cell functions through splicing events.^22^ CLTC, a coating protein involved in alveolar epithelial trafficking, participates in endocytosis and membrane vesicle trafficking at endosomes and lysosomes.^23^ Conversely, none of the BA-DEPs showed a correlation with F_ENO_ levels or Asthma Control Test scores (Fig 3, *F* and *G*).

For COPD-DEPs, 14-3-3 protein theta (YWHAQ) and galectin-related protein (LGALSL) were positively and negatively correlated with FEV_1_ value, respectively (Fig 3, *H* and *I*). YWHAQ, a member of the 14-3-3 family of proteins, acts as an adapter and chaperone, regulating various signaling pathways, including apoptosis.^24^ LGALSL, a member of the galectin family with sugar-binding domains, is involved in immune responses and cell-cell adhesion, although its precise functions are less well understood.^25^ Thus, the EBC proteome may have potential for noninvasive assessment of phenotypes and endotypes in both BA and COPD.

This is the first study to include a comprehensive analysis of the EBC proteome in both BA and COPD and apply DIA proteomics to EBC-EVs. We successfully identified the largest number of EBC proteins reported to date, surpassing even the number of those identified in another study using DIA proteomics on EBC without EV isolation.^26^ Additionally, this study is the first to analyze the identified EBC protein biomarkers in terms of their clinical relevance. Previous proteomics studies on BA have identified only peptide-level differences and have not explored altered pathways or clinical relevance.^27^

The results of this study suggest that diagnosis and phenotype and/or endotype estimation in BA and COPD can be conducted conveniently and noninvasively through breath sampling. Additionally, diagnosis using a multimarker approach that combines EBC biomarkers with F_ENO_, proteome-based patient clustering, or multiomics strategies—particularly metabolomics—could further enhance the utility of the EBC proteome. Finally, the effectiveness of EBC biomarkers could be further reinforced by combining them with established biomarkers, such as serum biomarkers.

This study has some limitations. First, the sample was small; large-scale validations are needed to confirm the utility of our biomarkers. Second, because the EV population can vary with the isolation method, our results may have limited reproducibility using other methods. The optimal methods for collection for EBC and processing and purification of EBC-EVs are not yet established. To apply biomarker proteins in EBC-EVs clinically, these challenges must be addressed to enhance the purity and recovery of EBC-EVs, ensuring consistent quantification.

In conclusion, EV isolation and in-depth nontargeted proteomics have enabled characterization of the EBC proteome in BA and COPD, leading to the identification of biomarkers that may reflect disease phenotypes and endotypes. The proteomic “breathprint” holds promise for noninvasive diagnosis and multifaceted evaluation. Integrating our proteome data with various omics data, clinical data, and conventional biomarkers could significantly enhance our understanding of the complex pathogenesis of BA and COPD.

For additional details regarding the study methods, see the Supplementary Methods (available in the Online Repository at www.jaci-global.org).

**Clinical implications**

The EBC-EV proteome offers potential for noninvasive diagnosis in BA and COPD. Combined with conventional clinical parameters and biomarkers, these novel biomarkers enhance diagnosis and understanding of disease pathogenesis.

## Disclosure statement

Supported by the 10.13039/501100001691Japan Society for the Promotion of Science Grants-in-Aid for Scientific Research Program (grants JP18H05282 [to A.K.] and JP19K08650 and 22K08283 [to Y.T.]), the Japan Agency for Medical Research and Development–10.13039/501100003382Core Research for Evolutional Science and Technology (research grant 22gm1810003h0001 [to A.K.]), the Cabinet Office of Japan Government for the Public/Private R&D Investment Strategic Expansion Program, the 10.13039/100020408Kansai Economic Federation (grant to A.K.), Mitsubishi Zaidan1 (grants to A.K.), the 10.13039/100008732Uehara Memorial Foundation (grant to Y.T.), the Nippon Foundation–10.13039/501100004206Osaka University Project for Infectious Disease Prevention, the MSD Life Science Foundation (grant to Y.S.), and the 10.13039/100019085Japanese Respiratory Foundation (grant to Y.T.).

Disclosure of potential conflict of interest: The authors declare that they have no relevant conflicts of interest.

## References

[1] Denton E., Price D.B., Tran T.N., Canonica G.W., Menzies-Gow A., FitzGerald J.M. (2021). Cluster analysis of inflammatory biomarker expression in the International Severe Asthma Registry. J Allergy Clin Immunol Pract.

[2] Kuruvilla M.E., Lee F.E., Lee G.B. (2019). Understanding asthma phenotypes, endotypes, and mechanisms of disease. Clin Rev Allergy Immunol.

[3] Aaron S.D., Vandemheen K.L., Whitmore G.A., Bergeron C., Boulet L.P., Côté A. (2024). Early diagnosis and treatment of COPD and asthma—a randomized, controlled trial. N Engl J Med.

[4] Fahy J.V. (2015). Type 2 inflammation in asthma—present in most, absent in many. Nat Rev Immunol.

[5] Loewenthal L., Menzies-Gow A. (2022). FeNO in asthma. Semin Respir Crit Care Med.

[6] Horváth I., Barnes P.J., Loukides S., Sterk P.J., Högman M., Olin A.C. (2017). A European Respiratory Society technical standard: exhaled biomarkers in lung disease. Eur Respir J.

[7] Yáñez-Mó M., Siljander P.R., Andreu Z., Zavec A.B., Borràs F.E., Buzas E.I. (2015). Biological properties of extracellular vesicles and their physiological functions. J Extracell Vesicles.

[8] Sinha A., Yadav A.K., Chakraborty S., Kabra S.K., Lodha R., Kumar M. (2013). Exosome-enclosed microRNAs in exhaled breath hold potential for biomarker discovery in patients with pulmonary diseases. J Allergy Clin Immunol.

[9] Koba T., Takeda Y., Narumi R., Shiromizu T., Nojima Y., Ito M. (2021). Proteomics of serum extracellular vesicles identifies a novel COPD biomarker, fibulin-3 from elastic fibres. ERJ Open Res.

[10] Purghè B., Manfredi M., Ragnoli B., Baldanzi G., Malerba B. (2021). Exosomes in chronic respiratory diseases. Biomed Pharmacother.

[11] Kawasaki T., Takeda Y., Kumanogoh A. (2024). Proteomics of blood extracellular vesicles in inflammatory respiratory diseases for biomarker discovery and new insights into pathophysiology. Inflamm Regen.

[12] Liang Y., Lehrich B.M., Zheng S., Lu M. (2021). Emerging methods in biomarker identification for extracellular vesicle-based liquid biopsy. J Extracell Vesicles.

[13] Pinkerton M., Chinchilli V., Banta E., Craig T., August A., Bascom R. (2013). Differential expression of microRNAs in exhaled breath condensates of patients with asthma, patients with chronic obstructive pulmonary disease, and healthy adults. J Allergy Clin Immunol.

[14] Kierbiedź-Guzik N., Sozańska B. (2023). The potential role of serum and exhaled breath condensate miRNAs in diagnosis and predicting exacerbations in pediatric asthma. Biomedicines.

[15] Yoo E.J., Kim J.S., Stransky S., Spivack S., Sidoli S. (2024). Advances in proteomics methods for the analysis of exhaled breath condensate. Mass Spectrom Rev.

[16] Welsh J.A., Goberdhan D.C.I., O'Driscoll L., Buzas E.I., Blenkiron C., Bussolati B. (2024). Minimal information for studies of extracellular vesicles (MISEV2023): from basic to advanced approaches. J Extracell Vesicles.

[17] Zhang Y., Jing Y., Qiao J., Luan B., Wang X., Wang L. (2017). Activation of the mTOR signaling pathway is required for asthma onset. Sci Rep.

[18] Konstantinidis T.G., Cassimos D. (2014). S100–a new biomarker in asthma?. Antioxid Redox Signal.

[19] Hwang S., Son S.W., Kim S.C., Kim Y.J., Jeong H., Lee D. (2008). A protein interaction network associated with asthma. J Theor Biol.

[20] Anger A.M., Armache J.P., Berninghausen O., Habeck M., Subklewe M., Wilson D.N. (2013). Structures of the human and Drosophila 80S ribosome. Nature.

[21] Lupi A., Della Torre S., Campari E., Tenni R., Cetta G., Rossi A. (2006). Human recombinant prolidase from eukaryotic and prokaryotic sources. Expression, purification, characterization and long-term stability studies. FEBS J.

[22] Pirmoradi S., Hosseiniyan Khatibi S.M., Zununi Vahed S., Homaei Rad H., Khamaneh A.M., Akbarpour Z. (2023). Unraveling the link between PTBP1 and severe asthma through machine learning and association rule mining method. Sci Rep.

[23] Royle S.J., Bright N.A., Lagnado L. (2005). Clathrin is required for the function of the mitotic spindle. Nature.

[24] Aitken A. (2006). 14-3-3 proteins: a historic overview. Semin Cancer Biol.

[25] UniProt Consortium, LGALSL - LGALSL (human) In UniProt: The universal protein knowledgebase. https://www.uniprot.org/uniprotkb/Q3ZCW2/entry.

[26] Ma L., Muscat J.E., Sinha R., Sun D., Xiu G. (2021). Proteomics of exhaled breath condensate in lung cancer and controls using data-independent acquisition (DIA): a pilot study. J Breath Res.

[27] Dompeling E., Jöbsis Q. (2011). Proteomics of exhaled breath condensate: a realistic approach in children with asthma?. Clin Exp Allergy.

