# Supplementary material for: Potential asthma biomarkers identified by nontargeted proteomics of extracellular vesicles in exhaled breath condensate
Source: J Allergy Clin Immunol Glob. 2025 Jan 30;4(2):100432. doi: 10.1016/j.jacig.2025.100432 (PMC11880581; doi:10.1016/j.jacig.2025.100432)
Supplement: Supplementary data [file mmc1.docx]

*Online Repository Materials*

Reina Hara, MD,^a^ Yoshito Takeda, MD, PhD,^a^ Takatoshi Enomoto, MD,^a^ Hanako Yoshimura, MD, PhD,^a^ Makoto Yamamoto, MD,^a^ Satoshi Tanizaki, MD, ^a^ Yuya Shirai, MD, PhD, ^a^ Takahiro Kawasaki MD, PhD, ^a^ Mana Nakayama, ^a^ Saori Amiya, MD,^a^ Yuichi Adachi, MD,^a^ Yoshimi Noda, MD,^a^ Takayuki Niitsu, MD,^a^ Ryuya Edahiro, MD,^a^ Moto Yaga, MD, PhD,^a^ Yuki Hosono, MD, PhD,^a^ Maiko Naito, MD, PhD,^a^ Kentaro Masuhiro, MD, PhD,^a^ Yujiro Naito, MD, PhD,^a^ Takayuki Shiroyama, MD, PhD,^a^ Kotaro Miyake, MD, PhD,^a^ Kiyoharu Fukushima, MD, PhD,^a^ Shohei Koyama, MD, PhD,^a^　Kota Iwahori, MD, PhD,^a^ Haruhiko Hirata, MD, ^a^ Izumi Nagatomo, MD, PhD,^a^ Yusuke Kawashima, PhD,^b^ Mari Nogami-Itoh, PhD,^c^ and Atsushi Kumanogoh, MD, PhD,^a,d-h^

^a^Department of Respiratory Medicine and Clinical Immunology, Osaka University Graduate School of Medicine, Suita, Osaka, Japan;

^b^Department of Applied Genomics, Kazusa DNA Research Institute, Kisarazu;

^c^Compound Library Screening Center Graduate School of Pharmacological Sciences, Osaka University Yamada-oka 1-6, Suita-City, Osaka 565-0871, Japan

^d^Department of Immunopathology, Immunology Frontier Research Center (WPI-IFReC), Osaka University, Suita, Osaka, Japan

^e^Center for Advanced Modalities and DDS (CAMaD), Osaka University, Osaka, Japan

^f^Center for Infectious Diseases for Education and Research (CiDER), Osaka University, Suita, Osaka, Japan

^g^Integrated Frontier Research for Medical Science Division, Institute for Open and Transdisciplinary Research Initiatives (OTRI), Osaka University, Suita, Osaka, Japan

^h^Japan Agency for Medical Research and Development–Core Research for Evolutional Science and Technology (AMED-CREST), Osaka University, Osaka, Japan

Corresponding author: Yoshito Takeda, MD, PhD, Department of Respiratory Medicine

and Clinical Immunology, Osaka University Graduate School of Medicine, 2-2 Yamada-Oka, Suita, Osaka, Japan. E-mail: [yoshito@imed3.med.osaka-u.ac.jp](mailto:yoshito@imed3.med.osaka-u.ac.jp).

**Methods**

***Participants***

Twenty-two participants were enrolled in this study, including eight individuals with asthma, five with chronic obstructive pulmonary disease (COPD), and nine healthy controls. Clinical data such as age, sex, smoking history, and other relevant information were collected. The asthma control test score was evaluated for participants with asthma, as detailed in Table I. Asthma was diagnosed following the Global Initiative for Asthma guidelines, and COPD was diagnosed according to the Global Initiative for Chronic Obstructive Lung Disease guidelines. Patients with asthma-COPD overlap, those treated with biologics or sequential systemic corticosteroids, individuals with comorbid pulmonary diseases, or those providing less than 500 μL of exhaled breath condensate (EBC) were excluded. Patients receiving oxygen therapy were also excluded due to difficulties in EBC collection. This research adhered to the Declaration of Helsinki for medical research involving human participants and was approved by the Osaka University Hospital ethics committee (No. 17148-6). Written informed consent was obtained from all participants.

***Collection of EBC***

The samples were collected at Osaka University Hospital from August 9, 2023 to April 4, 2024. The EBC was collected during normal breathing using an R-Tube (Respiratory Research Inc., USA), which consists of a collection tube and a pre-cooled covering sleeve. The samples were immediately frozen at−80°C. Before EBC collection, patients rinsed their mouths with water and wore a nose clip to prevent nasal breathing. The collection duration was 10–12 min.

***Isolation of extracellular vesicles (EVs)***

EVs were isolated using the phosphatidylserine (PS) affinity method^E1^ with the MagCapture Exosome Isolation Kit PS v.2 (FUJIFILM Wako, Tokyo, Japan), following the manufacturer’s instructions.

***DIA***

*Sample preparation*

Proteins in the EBC-EV samples were solubilized by adding 100 mM Tris (pH 8.0), 4% SDS, and 20 mM NaCl, followed by sonication using a closed sonicator. To reduce disulfide bonds, TCEP was added to the solubilized protein solution (20 µg protein) to a final concentration of 20 mM and incubated at 80°C for 10 min. For cysteine residue alkylation, iodoacetamide was added to a final concentration of 30 mM and incubated in the dark at room temperature for 30 min.

Next, a mixture of Sera-Mag SpeedBead carboxylate modified magnetic particles (with a 1:1 v/v ratio of hydrophilic to hydrophobic beads) was prepared and washed three times with distilled water. The final concentration was adjusted to 8 µg solids/µL. The alkylated sample was then combined with 20 µL of SP3 beads and ethanol (three times the volume of the sample) and mixed at room temperature for 20 min.

The beads were washed twice with 80% ethanol, and then 100 µL of 50 mM Tris-HCl (pH 8.0) was added and mixed. Proteins were digested overnight at 37°C with 500 ng of trypsin/Lys-C mix (Promega). After digestion, 20 µL of 5% TFA was added, and the sample was treated with a closed sonicator. The peptides were then desalted using a reversed-phase spin column (GL-Tip SDBB, GL Sciences) and dried using a centrifugal evaporator.

The dried peptides were reconstituted in 10 µL of 2% ACN-0.1% TFA and analyzed by LC-MS/MS.

***NanoLC-MS analysis***

The analysis was performed using an UltiMate 3000 RSLCnano LC System (Thermo Fisher Scientific). A total of 4 µL of peptides was injected. The column had an internal diameter of 75 µm and a length of 300 mm, packed with 1.7 µm C18 particles (100A, CoAnn Technologies), and was maintained at 60°C. The solvents used were 0.1% formic acid in distilled water (Solvent A) and 0.1% formic acid in 80% acetonitrile (Solvent B).

Mass spectrometry analysis was performed using an Orbitrap Exploris 480 MS (Thermo Fisher Scientific) in ESI positive mode. The total measurement time was 100 min, using data-independent acquisition (DIA). Data were acquired through alternating scan events: Full Scan (MS1) and DIA (MS2). For the full scan (MS1), spectra were collected within a mass range of 495 to 745 m/z with a resolution of 15,000. The automatic gain control (AGC) target was set to 3×10^6, and the maximum injection time was auto-adjusted. For the DIA (MS2), spectra were collected over a mass range of 200 to 1800 m/z with a resolution of 45,000. The AGC target was also set to 3×10^6, with an automatic maximum injection time. The normalized collision energy was set to 26%, and an isolation window of 4.0 m/z was used.

The MS data obtained were analyzed using DIA-NN under the following conditions for identification and quantification of proteins and peptides: protein sequence database (FASTA): Human UniProtKB/Swiss-Prot database (Proteome ID UP000005640, 20,591 entries, downloaded on March 7, 2023); FASTA digest for library-free search/library generation: On; deep learning-based spectra, RTs, and IMs prediction: On; digestion enzyme: trypsin; missed cleavages: 1; N-term M excision: On; C carbamidomethylation: On; peptide length range: 7–45; precursor charge range: 2–4; precursor m/z range: 490-750; fragment ion m/z range: 200–1800.

Using the DIA-NN 1.8.1 software, the identification and quantification of proteins were performed under the following conditions: mass accuracy: 10 ppm; fragment tolerance: 10 ppm; precursor FDR: ≤1%; protein FDR: ≤1%; unrelated runs: On; use isotopologues: On; heuristic protein inference: On; no shared spectra: On; protein inference: genes; neural network classifies: single pass mode; quantification strategy: robust LC (high precision); cross run normalization: RT dependent; library generation: smart profiling; speed and RAM usage: optimal results.

***DIA data analysis***

Quantitative values were log-transformed using the Perseus software platform.^E2^ Proteins with ≥50% missing values across all samples were excluded. Missing values were addressed using the randomized assignment method.

***Statistical analyses***

T-tests to identify differentially expressed proteins (DEPs) in asthma (BA-DEPs) and DEPs in COPD (COPD-DEPs) were performed using R Statistical Software (version 4.3.1; 2023-06-16). Welch’s t-test, Kruskal–Wallis test, Mann–Whitney U test, and Pearson chi-square test were conducted using Microsoft Excel (version 16.16.27(201012)). Spearman’s rank correlation tests were performed using GraphPad Prism version 10.0. A P-value of <.05 was considered statistically significant.

***Nanoparticle tracking analysis***

Particle numbers and size distribution of EVs were analyzed using NanoSight Pro (Malvern Panalytical, Worcestershire, UK)^E3^. Briefly, nanoparticle tracking analysis (NTA) was conducted on isolated EVs, with all events recorded in a video for subsequent analysis using NTA software. The Brownian motion of particles was tracked between frames, and particle size was calculated using the Stokes–Einstein equation.

***Transmission electron microscopy of EVs***

Samples of EVs (50 μL) were adsorbed onto a collodion-coated grid for 10 min. The EVs were then fixed with 2% paraformaldehyde and incubated with an anti-CD63 antibody (MX-49.129.5, sc-5275; Santa Cruz Biotechnology, Dallas, TX, USA). Immunoreactive EVs were visualized using an anti-mouse IgG antibody (EMGMHL10; B BI solutions, Cardiff, Wales, UK) preabsorbed with 10 nm gold particles.

***Pathway enrichment analysis***

Canonical pathway analysis and diseases and function analysis were performed using Ingenuity Pathway Analysis software (IPA®, ver. Spring 2024, Qiagen Inc., Redwood City, CA, USA).

***Protein-protein interactions***

To identify biologically relevant molecular networks and pathways in the proteome, Ingenuity Pathway Analysis was employed. For BA-DEPs or COPD-DEPs, interactions were examined for the pathways obtained from the disease and function analysis, and network generation was performed. Each network was weighted by scoring, and protein-protein interactions were plotted for the top networks.

***References***

E1. Nakai W, Yoshida T, Diez D, Miyatake Y, Nishibu T, Imawaka N, et al. A novel affinity-based method for the isolation of highly purified extracellular vesicles. Sci Rep 2016;6:33935.

E2. Tyanova S, Temu T, Sinitcyn P, Carlson A, Hein MY, Geiger T, et al. The Perseus computational platform for comprehensive analysis of (prote)omics data. Nat Methods 2016;13:731-40.

E3. Yoshioka Y, Kosaka N, Konishi Y, Ohta H, Okamoto H, Sonoda H, et al. Ultra-sensitive liquid biopsy of circulating extracellular vesicles using ExoScreen. Nat Commun 2014;5:3591.
